# Combating dengue using male *Wolbachia-*infected mosquitoes–lessons from Singapore's experience

**DOI:** 10.1016/j.lanwpc.2026.101841

**Published:** 2026-03-26

**Authors:** Jue Tao Lim, Vernon J. Lee, Cheong Huat Tan, Chee-Seng Chong, Kathyrn Vasquez, Lee Ching Ng

**Affiliations:** a Lee Kong Chian School of Medicine, Nanyang Technological University, Singapore; b Communicable Diseases Agency, Singapore; c Environmental Health Institute, National Environment Agency, Singapore; d Saw Swee Hock School of Public Health, National University of Singapore, Singapore; e School of Biological Sciences, Nanyang Technological University, Singapore

Urgency for *Aedes*-borne disease control is warranted, warming temperatures and an increasingly urban global population are set to further expand the geographical range and intensity of dengue transmission. Low herd immunity in newly at-risk regions endangers many to dengue. Preparedness via innovative environmental public health interventions must be undertaken to meet this threat, as conventional methods have not mitigated dengue historically. Yet, achieving dengue control through vector control remains difficult. At present, spatial repellents^1^ and *Wolbachia*-mediated incompatible insect technique with sterile insect technique (IIT-SIT)^2^ are the only tools which have empirically demonstrated a concurrent reduction in dengue risk and vector abundance.

In Singapore, Project *Wolbachia,* which started in 2016, encompasses the deployment of *Wolbachia*-mediated IIT-SIT via releasing solely male *Wolbachia*-infected *Aedes aegypti*. These mosquitoes, incapable of biting, mate with wildtype females that produces non-viable eggs and reducing mosquito populations. Releases will cover over 50 per cent of all households by 2026, or approximately 3.2 million residents.^3^ It is one of the few new innovations in the fight against dengue. As of date, it is the only trial of IIT-SIT which has been reported to reach operational status^4^ and yielded evidence on an estimated 80–90% reduction in vector populations and more than 70% reduction in the risk of contracting dengue.^2^ IIT-SIT also confers a short-range spillover benefits with a 45% reduction in dengue risk in adjacent areas.^5^ Since the start of the programme, dengue cases reported in 2025 was the lowest since 2018.

Singapore's programme may be the largest reported trial and roll-out of a vector-control tool for operational dengue control. The intervention's efficacy offers hope for high-density urban areas vulnerable to outbreaks of dengue and perhaps other *Aedes*-mosquito-borne diseases. As with any vector control tool, IIT-SIT benefits take time (typically 3–12 months) to see full epidemiological effect, requires consistent releases for suppression and a sizeable release area to confer substantial public health gains. The approach also requires careful entomological surveillance, ensuring that no mistaken releases of fertile females occur, and an acute understanding of local, social and environmental dynamics. It is also uncertain how these efficacy estimates translate to other epidemiological settings. These complexities emphasise the importance of careful planning for global replication.

Factors driving the success of implementing IIT-SIT go beyond the endpoints of entomological and epidemiological efficacy. Firstly, IIT-SIT is a novel technology and requires strong support from all levels of the population. Political and government support is needed to ensure sustainable resources are available for this long-term endeavour. Local support together with community engagement are necessary to build acceptance and prevent disinformation from derailing efforts.^6^^,^^7^ Public education plays a vital role in reinforcing sustainable mosquito control practices, reducing reliance on chemical interventions, and embedding these efforts within a comprehensive integrated pest management strategy. Secondly, IIT-SIT is not a panacea and must be paired with other sustainable solutions. In Singapore, decades of empowering government agencies and community to take ownership of environmental management had drastically reduced baseline vector population to enhance the entomological effectiveness and cost-effectiveness of IIT-SIT. Low baseline wild-type vector population and continued source reduction in the community renders it possible to suppress wild-type populations with far fewer *Wolbachia*-infected male mosquitoes.^5^ Vaccines, where available, may also complement the strategy and will need to be further studied. Thirdly, the technology requires sustained investment, institutional memory, and continuity of expertise, but public health innovations rarely fit neatly within short grant funding cycles. Substantial long-term investment must be committed, buffered by strong political and leadership support. Lastly, a phased approach in implementing the technology is important to enable gradual progress in size and complexity, enabling effective troubleshooting and design of the subsequent phases based on concrete evidence. In Singapore, each trial phase is conducted with a clear objective, with full intent to further expand the intervention. They yielded valuable evidence on efficient release strategies, the competitiveness of *Wolbachia*-infected mosquitoes,^8^ and its epidemiological^9^ and entomological effectiveness,^10^ cost-effectiveness^11^ and operational business model. This is relevant in any new roll-out because of different local contexts.

To facilitate adoption in other cities, additional groundwork is necessary: ensuring stability of the *Wolbachia* infection, characterising the level of cytoplasmic incompatibility that *Wolbachia*-infected male mosquitoes can induce in wild-type mosquito populations; ensuring the ability for these mosquitoes to sexually compete and traverse areas intended for implementation; triangulating the baseline level of mosquito abundance to understand the absolute numbers required for effective suppression, and forward plan the resources needed for sustained implementation of the intervention; and engagement across the population to educate and garner feedback on the technology to improve implementation.

## Declaration of interests

None.
